# Associations between gut microbiota and genetic risk for rheumatoid arthritis in the absence of disease: a cross-sectional study

**DOI:** 10.1016/S2665-9913(20)30064-3

**Published:** 2020-06-25

**Authors:** Philippa M Wells, Adewale S Adebayo, Ruth C E Bowyer, Maxim B Freidin, Axel Finckh, Till Strowig, Till Robin Lesker, Deshire Alpizar-Rodriguez, Benoit Gilbert, Bruce Kirkham, Andrew P Cope, Claire J Steves, Frances M K Williams

**Affiliations:** a Department of Twin Research and Genetic Epidemiology, King's College London, London, UK; b Centre for Rheumatic Diseases, King's College London, London, UK; c Centre for Inflammation Biology and Cancer Immunology, King's College London, London, UK; d Division of Rheumatology, Geneva University Hospital, Geneva, Switzerland; e Department of Microbial Immune Regulation, Helmholtz Centre for Infection Research, Braunschweig, Germany; f Hannover Medical School, Hannover, Germany; g Department of Rheumatology, Guy's and St Thomas’ NHS Trust, London, UK; h Department of Ageing and Health, St Thomas’ Hospital, London, UK

## Abstract

**Background:**

Rheumatoid arthritis is a chronic inflammatory autoimmune disease that is associated with reduced life expectancy. The disease is heritable and an extensive repertoire of genetic variants have been identified. The gut microbiota might represent an environmental risk factor for rheumatoid arthritis. We aimed to assess whether known rheumatoid arthritis risk alleles were associated with the gut microbiota in a large population who do not have rheumatoid arthritis.

**Methods:**

In this cross-sectional study done in the UK and Switzerland, we used genotyping and microbiota data from previous studies of the TwinsUK cohort, excluding participants who had ever had a diagnosis of rheumatoid arthritis, as well as their unaffected co-twins. We used blood samples for genotyping and stool samples for the assessment of the gut microbiota. We generated a polygenic risk score (PRS) for rheumatoid arthritis in 1650 TwinsUK participants without the disease, based on 233 GWAS-identified single nucleotide polymorphisms associated with rheumatoid arthritis. We validated the PRS using logistic regression against rheumatoid arthritis diagnosis in 2686 UK Biobank individuals with a confirmed diagnosis of rheumatoid arthritis. Amplicon sequence variants (ASVs) were generated from 16S rRNA gene sequencing of stool samples and assessed for association with the PRS for rheumatoid arthritis. We validated the findings in an independent sample comprised of first-degree relatives of patients with rheumatoid arthritis from the SCREEN-RA cohort. Differential abundance of ASVs present in more than 5% of samples, grouped by ASV taxon annotation, against the rheumatoid arthritis PRS as a continuous variable was assessed using fixed-effects covariates. To account for multiple testing, the false discovery rate calculation was applied to all p values to generate q values, with a significance threshold of 0·05 determined a priori.

**Findings:**

We found that presence of *Prevotella* spp were positively associated with the rheumatoid arthritis PRS in TwinsUK participants (q<1 × 10^−7^). This finding was validated in SCREEN-RA participants (n=133) carrying established shared epitope risk alleles (q=0·0011). We also found an association between *Prevotella* spp and presence of preclinical rheumatoid arthritis phases (q=0·021).

**Interpretation:**

*Prevotella* spp in the gut microbiota are associated with the rheumatoid arthritis genotype in the absence of rheumatoid arthritis, including in individuals at high risk of developing rheumatoid arthritis. Our findings suggest that host genotype is associated with microbiota profile before disease onset.

**Funding:**

Versus Arthritis.

## Introduction

Rheumatoid arthritis is a debilitating chronic autoimmune condition, associated with reduced life expectancy. The cause of rheumatoid arthritis has a substantial genetic component, with heritability estimated at 65%.^1^ Known environmental risk factors include periodontal disease, tobacco smoking, and diet, and these appear to trigger disease onset in genetically susceptible individuals.^2^ An additional rheumatoid arthritis risk factors is the mucosal commensal microbiota. Extensive cross-talk exists between microbiota and the host, starting early in life with the development of a normal immune system; microbiota might be implicated in the development of rheumatoid arthritis.

The gut lumen holds most of the commensal microbiota, and has intimate proximity to both the immune system via the gut-associated lymphoid tissue, and the systemic circulation. A key gut microbiota association in patients with rheumatoid arthritis is a relative increase in the abundance of *Prevotella* spp,^3^ particularly *Prevotella copri* (*P copri*), which presents early in the course of rheumatoid arthritis.^4^, ^5^ *P copri* is also positively associated with clinical parameters in patients with rheumatoid arthritis, further supporting its pathophysiological relevance to the disease.^5^ In addition to promoting disease activity, the gut microbiota might also influence the response to treatment of patients with rheumatoid arthritis.^5^, ^6^ The gut microbiota therefore represents a potential therapeutic target in patients with rheumatoid arthritis, both for the modulation of disease and for improving the response to established therapeutics.

**Research in context**

**Evidence before this study**

We searched Google Scholar using the search terms “rheumatoid arthritis microbiome”; “rheumatoid arthritis Prevotella”; “microbiome heritability”; “microbiome, genetics rheumatoid arthritis”; and “rheumatoid arthritis genetic aetiology”. We included articles published in English between Jan 1, 1990, and Oct 1, 2019. The reference lists of identified papers were further used to identify relevant literature. *Prevotella copri* has been shown to be increased within the gut microbiota of patients with rheumatoid arthritis, predominantly those with early disease, before treatment is initiated. Prevotellaceae family members have also been shown to be higher in patients with pre-clinical rheumatoid arthritis than in controls. *P copri* is posited to be an inflammatory driver, contributing to rheumatoid arthritis pathology by promoting a pro-inflammatory cytokine milieu.

**Added value of this study**

This study is, to our knowledge, the first to show that, in a large cohort, carrying genetic risk factors for rheumatoid arthritis is associated with a higher abundance of *Prevotella* spp in the absence of any form of rheumatoid arthritis.

**Implications of all the available evidence**

Within the gut microbiota, *Prevotella* spp, including but not limited to *P copri*, are of interest in the pathogenesis of rheumatoid arthritis. Our findings suggest that any potential causal role of *Prevotella* spp occurs early in disease development, and therefore targeting of the preclinical stage of rheumatoid arthritis should be explored in future studies.

Identifying how genetic and environmental risk factors for rheumatoid arthritis interact with one another might shed light on the underlying biology of the disease. The influence of host genetic factors on the microbiota in rheumatoid arthritis remains somewhat unexplored. An important influence is highly plausible: host genetics shape the biochemical and immune environment in which the microbiota reside, and furthermore the cumulative influence of the genetic risk loci in rheumatoid arthritis is predominantly mediated by immune pathways.^2^ Although several factors influence microbiota composition, host genetic factors account for a considerable proportion of variance, with some taxa being 40% heritable.^7^

We aimed to investigate whether genetic risk for rheumatoid arthritis was associated with the composition of the gut microbiota in the absence of clinical disease.

## Methods

### Study design and participants

**Figure 1:**
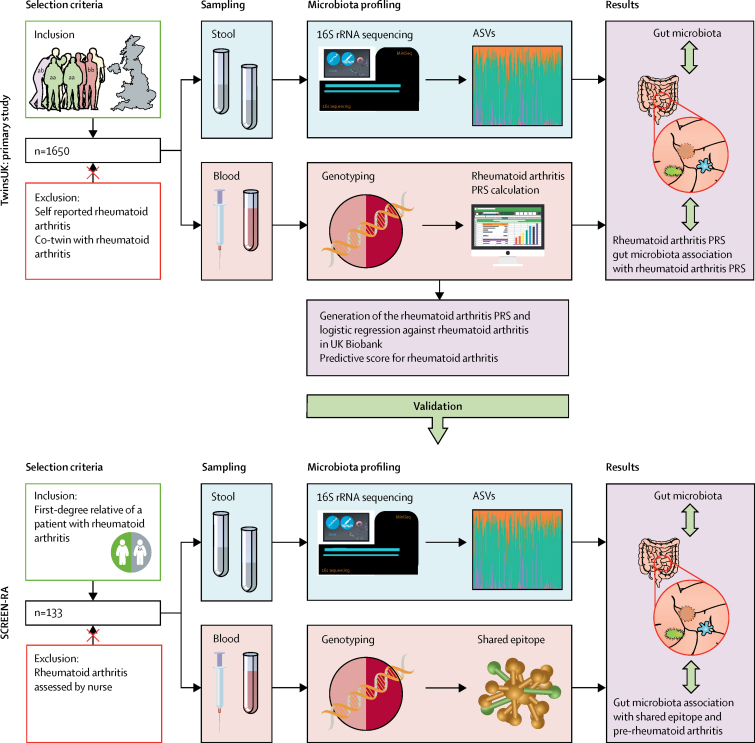
Schematic representation of the study design The sampling, microbiota profiling, and genotyping were previously performed (blue and pink panels).^8^ The purple panels indicate analyses done in the current study. The TwinsUK cohort is led by researchers at King's College London, located at St Thomas Hospital NHS Foundation Trust, and comprises adult twins who are resident in the UK. SCREEN-RA is a Swiss multi-centre cohort, which comprises first-degree relatives of patients with rheumatoid arthritis. ASV=amplicon sequence variant. PRS=polygenic risk score.

This cross-sectional study was done in the UK and Switzerland, and included participants from the TwinsUK (primary) and SCREEN-RA (validation) cohorts. To isolate the genetic influence on disease from the pathophysiology and treatment influence of established disease, TwinsUK participants with rheumatoid arthritis and their co-twins were excluded from the study. We used genotyping and microbiota data from 1650 eligible TwinsUK participants who had contributed blood and stool samples (figure 1). Genetic risk for rheumatoid arthritis in TwinsUK participants was captured using polygenic risk scoring. A polygenic risk score (PRS) for rheumatoid arthritis was generated and validated using logistic regression against rheumatoid arthritis diagnosis in UK Biobank participants, with 2686 confirmed rheumatoid arthritis cases, and applied to rheumatoid arthritis-unaffected participants in TwinsUK. We then calculated the association between the PRS for rheumatoid arthritis and composition of the gut microbiota.

Ethics approval was granted by the St. Thomas’ Hospital Research Ethics Committee. Following the restructure and merging of the research ethics committee, subsequent amendments were approved by the National Research Ethics Service (NRES) Committee London–Westminster (TwinsUK reference EC04/015); approval for the use of microbiota samples was granted by the NRES Committee London–Westminster (The Flora Twin Study reference 12/LO/0227).

Participants included in the study were members of the TwinsUK cohort, the largest UK registry of adult twins.^9^ We excluded participants who reported ever having had a diagnosis of rheumatoid arthritis, as well as their unaffected co-twin. This strategy allows for isolation of rheumatoid arthritis genetic factors and circumvents the important issues of confounding by rheumatoid arthritis disease and its treatment—thereby achieving a human model of the genetic association with the microbiota in rheumatoid arthritis. The TwinsUK registry is demographically well suited to the study of rheumatoid arthritis (most participants are female and older); as such, we deemed that further study-specific inclusion criteria were not necessary. TwinsUK participants comprised 93% women, with a median age of 63 years (table).

**Table:** Participant demographics

		**TwinsUK: rheumatoid arthritis unaffected (n=1650)**	**SCREEN-RA**	
			Pre-rheumatoid arthritis (n=83)	FDR controls (n=50)
Age, years		63 (56–69)	58 (50–66)	55 (47–62)
Sex				
	Women	1535 (93%)	74 (89%)	39 (78%)
	Men	115 (7%)	9 (11%)	11 (22%)
BMI		25 (23–29)	24 (22–27)	24 (22–27)
ACPA positive		9* (2%)	38 (46%)	0
Rheumatoid factor positive		35*(7%)	28 (34%)	0
Shared epitope positive		..	42 (51%)	32 (64%)
ACPA and rheumatoid factor positive		1 (<1%)	6 (7%)	0
Current smoker		567 (34%)	16 (19%)	11 (22%)
Antibiotic use within the past month		66 (4%)	..	..
Caucasian; northern European		1650 (100%)	83 (100%)	50 (100%)
Swollen joints		..	1 (0–3)	0 (0–1)
Tender joints		..	1 (0–2)	0 (0–1)

Data are median (IQR), or n (%). ACPA positivity was defined as a concentration of greater than 5 uL/mL. Rheumatoid factor positivity was defined as a concentration of greater than 15 uL/mL. FDR=false discovery rate. BMI=body-mass index. ACPA=anti-citrullinated protein antibody.

* Serum samples analysed for 500 TwinsUK participants, including those with highest polygenic risk scores.

To further confirm the results from TwinsUK, an analysis of genetic predisposition to rheumatoid arthritis and microbiota association was done in participants of SCREEN-RA, a Swiss multi-centre cohort of first-degree relatives of patients with rheumatoid arthritis, who themselves are unaffacted by the disease (figure 1).^8^ These relatives share on average 50% of their genotype with their rheumatoid arthritis-affected relative, and thus have a higher genetic risk of rheumatoid arthritis than do those in the general population. Within SCREEN-RA participants (n=133), the subset of patients with preclinical rheumatoid arthritis (n=83) were identified using the European League Against Rheumatism (EULAR) terminology for preclinical phases of rheumatoid arthritis,^8^, ^10^ and matched with 50 controls who were also first-degree relatives of patients with rheumatoid arthritis but who did not have preclinical arthritis. Briefly, preclinical rheumatoid arthritis was defined on the basis of serum positivity for anti-citrullinated protein antibody (ACPA) or rheumatoid factor, or symptoms and signs associated with possible rheumatoid arthritis with or without undifferentiated arthritis.^8^, ^10^ Patients with a diagnosis of rheumatoid arthritis were excluded from the analysis and all participants were examined by a trained nurse. SCREEN-RA participants had previously been genotyped for the *HLA-DRB1* shared-epitope risk allele for rheumatoid arthritis, the strongest established genetic association with disease. We examined the gut microbiota composition in relation to the shared-epitope genotype and presence or absence of pre-rheumatoid arthritis in this cohort. Of SCREEN-RA participants, 74 (89%) were women and the median age was 57 years (table). Participants provided written informed consent.

### Procedures

#### Genotyping—TwinsUKcohort

We used sequencing data which had been previously generated as part of the TwinsUK cohort, which is led by the Department of Twin Research and Genetic Epidemiology, King's College London (London, UK). Briefly, blood samples from TwinsUK participants obtained at the clinical visit were used to identify genotype using the Illumina HumanHap300 BeadChip and the Illumina HumanHap610 QuadChip (Illumina, Cambridge, UK). Non-genotyped variants were imputed using 1000 Genomes and Haplotype Reference Consortium reference panels.^9^ Testing was undertaken by Affinity Biomarker Labs using the Siemens Centaur XP Anti-Cyclic-C Peptide (Siemens Health Engineers, Frimley, UK). Seropositivity was defined as more than 5 U/mL.

#### PRS for rheumatoid arthritis in TwinsUK

A PRS to predict rheumatoid arthritis in unaffected TwinsUK participants was calculated and its association determined with composition of the gut microbiota. The PRS assigns an individual a single numerical value for risk of disease conferred by genetic factors.^11^ The NCBI database of GWAS summary statistics for rheumatoid arthritis was used to identify 233 published single nucleotide polymorphisms (SNPs) associated with rheumatoid arthritis at genome-wide significance (p=5 × 10^−8^), of which 117 had been replicated across studies (appendix p 3).^12^ European ancestry was included as a study inclusion criterion, ensuring ethnic concordance with TwinsUK (ie, ethnicities other than white were excluded).^9^, ^13^ The PRS was tested for a predictive value for rheumatoid arthritis in 6776 participants from UK Biobank, including 2686 patients with rheumatoid arthritis and 4090 unselected controls with the chronologically closest participant identification numbers. Diagnosis of rheumatoid arthritis in UK Biobank participants had been made using hospital episode statistics data supplied by NHS Digital. All identified rheumatoid arthritis patients were included. We deemed that no exclusion criteria were necessary; the UK Biobank participants and the TwinsUK cohort had similar racial demographics. Logistic regression of 2686 patients with rheumatoid arthritis and unselected controls against PRS, adjusting for age, sex, and smoking history, was applied*.* Standardised coefficients are reported.

Risk allele dosage of SNPs present within TwinsUK was extracted using PLINK (version 1.9). Of the SNPs identified in the literature, 227 were available in TwinsUK. Pruning was applied to account for linkage disequilibrium.^14^ Missing allele dosages were imputed and replaced with the mean value across the respective SNPs. The risk allele dosage was multiplied by the SNP–rheumatoid arthritis association effect size, to produce a weighted PRS.^11^

#### Microbiota profiling—TwinsUK

We used data that had been previously generated as part of the TwinsUK cohort. Briefly, microbiota composition of stool samples was assessed using the 16S rRNA marker gene, with sequencing of the V4 variable region using barcoded primers (F515/R806). Samples were processed as previously described.^7^ Briefly, faecal samples were collected during clinical visits or were posted in sealed ice packs and frozen on arrival at the lab at −80°C. Stool samples were sent as 35 batches on dry ice to Cornell University, NY, USA, where DNA was extracted and sequenced on an Illumina MiSeq platform (Illumina, San Diego, CA, USA).^7^

16S sequences were demultiplexed in QIIME. Amplicon sequence variants (ASVs) were generated using the DADA2 package in R.^15^ Sequences were trimmed, error was estimated within the forward and reverse reads for each sample, and the ASV algorithm was applied to infer the original biological sequence. Forward and reverse reads were joined. Chimeras were removed and the total dataset was merged, followed by performing taxonomic assignment using SILVA version 1.3.2.^16^ Samples with a sequencing depth of less than 10 000 reads were excluded. A phylogenetic tree was generated using the Phangorn R package. Alpha diversity was calculated on untrimmed ASV tables using four measures: Shannon index, Simpson index, observed ASVs, and Faith's phylogenetic diversity. For the taxonomic analysis, ASVs were grouped according to taxon annotation. Taxonomic assignment using the SILVA database allows for a higher level of differentiation than other databases because in some instances the genus of ASVs can be annotated according to the prediction of species group.^17^ These annotations were preserved because they provide more information regarding taxon assignment than genus annotation alone. In this way, *Prevotella*-annotated ASVs might be accurately further differentiated using 16S data, which has been a methodological challenge previously.

#### ACPA testing–TwinsUK

500 TwinsUK participants, including 250 (50%) participants with high genetic risk (PRS within the upper quartile) were tested for ACPA seropositivity. Testing was done by Affinity Biomarker Labs, using Siemens Centaur XP Anti-cyclic-C peptide kit (Siemens Health Engineers, Frimley, UK). ACPA seropositivity was defined as more than 5 U/mL.

#### Phylogenetic and community relationship within the Prevotellaceae family

To investigate the *Prevotella* spp associations that were identified further, the phylogenetic and community relationships between the two implicated groups of ASVs (*Prevotella_7* and *Prevotella_9*) were explored. For these methods, grouping ASVs by taxon annotation was not appropriate and ASVs were considered as singular units. To examine the microbial ecological community relationships, ASVs were grouped into compositional clusters, or balances. Briefly, ASVs that were present in at least 10% of individuals were correlated and transformed as implemented by Morton and colleagues.^18^ This method creates clades in which interacting species are closer neighbours in a clade than loosely related ones are.

#### Shared epitope genotyping—SCREEN-RA cohort

As a validation step, we examined the gut microbiota composition in first-degree relatives of rheumatoid arthritis patients (SCREEN-RA cohort) in relation to shared epitope positivity, and a pre-disease state as defined by clinical rheumatoid arthritis parameters.^8^, ^10^

We used sequencing data which had been previously generated as part of a previous study of the SCREEN-RA cohort. We contacted the corresponding author of Alpizar-Rodriguez and colleagues’ study^8^ who co-leads the SCREEN-RA cohort, and they agreed to share their SCREEN-RA cohort data. Briefly, Alpizar-Rodriguez and colleagues^8^ obtained blood from SCREEN-RA participants and controls. DNA was extracted using a modification of the salt-out technique (Nucleon TM, Scotlab, Glasgow, UK). *HLA-DRB1* shared epitope polymorphism was assessed by reverse PCR (sequence-specific oligonucleotide primers hybridisation and by PCR) sequence-specific primers (SSP) using commercial reagents validated at the Swiss National Reference Laboratory for Histocompatibility. The method discriminates all major subtypes in different allele groups within the DRB1*04 genotype. *HLA-DR1*, *DR4*, and *DR14* alleles that were negative for the shared epitope 70–74 motif were also discriminated. In a second step, the shared epitope-positive typing ambiguities were analysed by PCR-SSP to find the final 4-digit typing result.

#### Microbiota profiling—SCREEN-RA cohort

Gut microbiota 16S rRNA data was collected as previously described.^8^ Briefly, the DNA Genotek OMNIgene Gut Stool Microbiome Kit (DNA Genotek, Ottawa, ON, Canada) was used to collect, store, and ship the stool samples. After sample processing and DNA extraction, the V4 region of the 16S rRNA gene was amplified using barcoded primers (F515/R806), and sequenced on an Illumina MiSeq (Illumina, San Diego, CA, USA), with ASVs generated as per the TwinsUK cohort to ensure compatibility.

### Statistical analysis

Linear mixed-effects models were used to determine association between rheumatoid arthritis PRS and alpha diversity, using alpha diversity as a response variable to the rheumatoid arthritis PRS as a continuous variable. Modelling was performed using the lme4 package in R,^19^ with fixed effect covariates age, body-mass index, and sequencing depth and technical covariates as random effects. Standardised coefficients were reported.

Differential abundance of ASVs present in more than 5% of samples, grouped by ASV taxon annotation, against the rheumatoid arthritis PRS as a continuous variable was assessed using the DESeq2 R package,^20^ using fixed-effects covariates. To account for multiple testing, the false discovery rate calculation was applied to all p values to generate q values, with a significance threshold of 0·05 determined a priori.

To examine the phylogenetic relationship, the phylogenetic tree was subsetted to Prevotellaceae and visualised using the Phyloseq R package.^21^

Differential abundance of taxa in the gut microbiota in association with preclinical rheumatoid arthritis and shared epitope positivity was assessed against all genus present in greater than 5% of samples using the DESeq2 R package.^20^ Biological covariates were not required to adjust for because these factors were not statistically different between the case and control groups (appendix p 1). We used the same method as Alpizaar-Rodriguez and colleagues,^8^ with the advancement that ASVs and DESeq2 methods were used.

### Role of the funding source

The funders of the study had no role in study design, data collection, data analysis, data interpretation, or writing of the report. PMW, ASA, MBF, RCEB, CJS, and FMKW had access to the raw data. The corresponding authors had full access to all of the data and the final responsibility to submit for publication.

## Results

1650 TwinsUK participants without rheumatoid arthritis were included, most of whom were women (table). The PRS was normally distributed in the TwinsUK sample with patients with rheumatoid arthritis and their twin siblings excluded (Shapiro-Wilk normality test p=0·24). Logistic regression of the PRS in 6776 UK Biobank participants, of which 2686 (39·6%) had a diagnosis of rheumatoid arthritis according to hospital episode statistics, confirmed that the score is predictive of rheumatoid arthritis (odds ratio per SD 1·34; p=4·17 × 10^−8^).

Individuals with a high PRS in TwinsUK did not show rheumatoid arthritis seropositivity: only nine (2%) of 500 individuals in the TwinsUK sample who had serum available were positive for ACPA, defined as more than 5 U/mL (table), and were distributed similarly across genetic risk groups, by PRS quartile (high 2:93; low 7:398).

We investigated whether genetic risk of rheumatoid arthritis was associated with alpha (within sample) diversity. We found no detectable association between PRS for rheumatoid arthritis and Shannon index (p=0·76), Simpson index (p=0·41), observed ASVs (p=0·60) or Faith's phylogenetic diversity (p=0·65; appendix p 2). Alpha diversity measures indicate microbial density (observed ASVs) and distribution, in which the distribution of more abundant versus less abundant taxa is assessed, or both of these factors (Shannon index, Simpson index). Faith's phylogenetic diversity is based on the phylogenetic distance between taxa in a sample.

**Figure 2:**
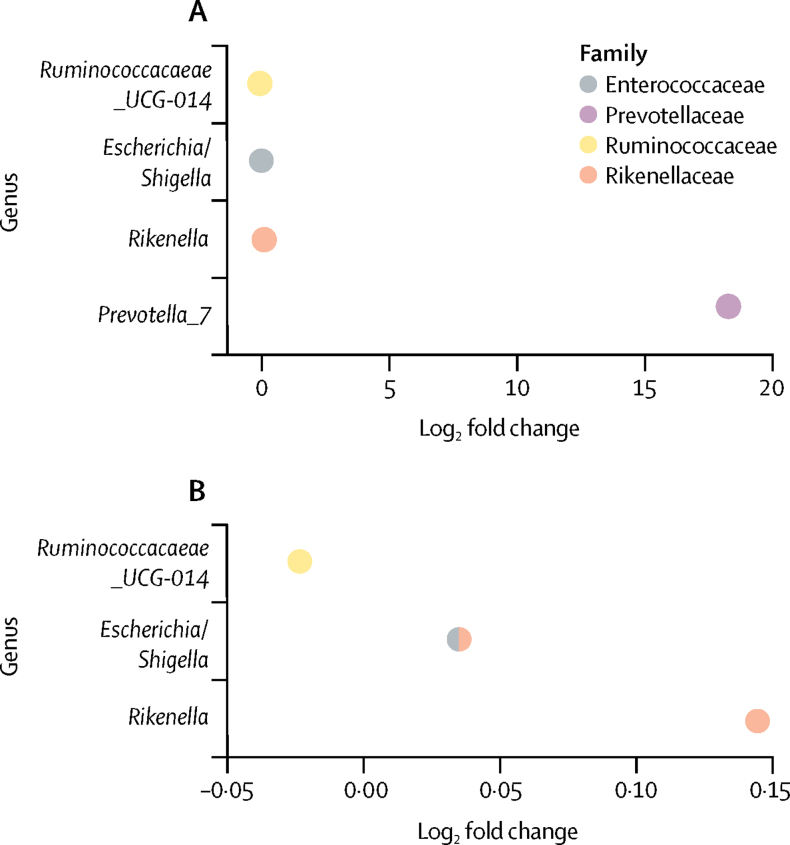
Differential abundance of the gut microbiota per unit increase in rheumatoid arthritis PRS in TwinsUK participants Positive log-fold change of taxa indicates a positive association with the rheumatoid arthritis PRS. *Prevotella_7* was the strongest association, and positively associated with the rheumatoid arthritis PRS (q<1 × 10^−7^). Because of the scale and comparative difference in log-fold change in *Prevotella*, this taxon was excluded (B) to allow visualisation of the three other associations. Other taxa associations within the gut microbiota were *Ruminococcaceae_UCG-14* (q=0·045), *Rikenella* (q=0·018), and *Shigella* (q=0·018). PRS=polygenic risk score.

The gut microbiota were taxonomically assessed for association with PRS for rheumatoid arthritis following a non-targeted approach. *Prevotella* ASVs were grouped together according to predicted species and given a numerical designation in the SILVA database. Within these groups, *Prevotella_9* was predicted to be *P copri,* whereas *Prevotella_7* was annotated to multiple *Prevotella* spp, with low sequence divergence. Of all 172 microbial taxa that were present in the gut (stool) microbiota of more than 5% of participants, *Prevotella_7* had the strongest taxon association with the PRS for rheumatoid arthritis (18-fold log base 2 higher differential abundance; q<1 × 10^−7^; figure 2). No additional *Prevotella* assocations with the PRS for rheumatoid arthritis were found in the TwinsUK cohort.

We sought to confirm our TwinsUK findings in the SCREEN-RA cohort by analysing the association between the main genetic risk factor for rheumatoid arthritis—the HLA-DRB1 shared epitope—and the gut microbiota. We also investigated the association of the gut microbiota with preclincal stages of rheumatoid arthritis in SCREEN-RA participants.

**Figure 3:**
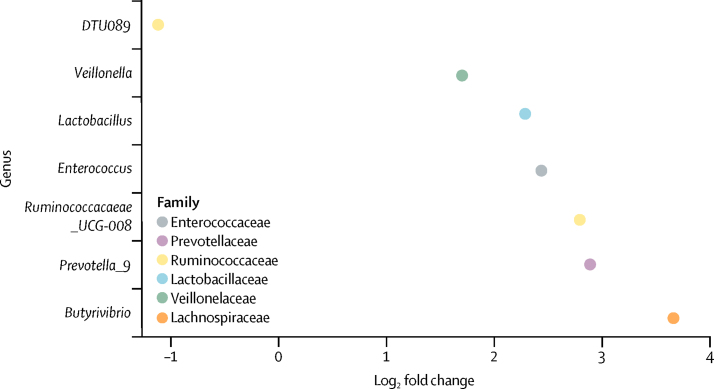
Differential abundance of the gut microbiota in patients with preclinical rheumatoid arthritis compared with unaffected controls in the SCREEN-RA cohort *Prevotella_9* was associated with preclinical rheumatoid arthritis (q=0·021). We found six additional genus group associations: *Lactobacillus* (q=0·0003), *Butyrivibrio* (q=0·018), *Ruminococcaceae*_*UCG*-*D08* (q=0·018), *Enterococcus* (q=0·018), *DTU089* (q=0·042), and *Veilllonella* (q=0·042).

**Figure 4:**
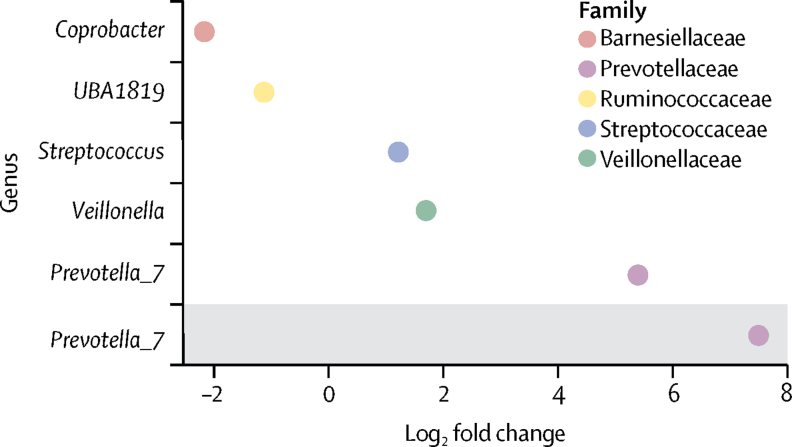
Differential abundance of the gut microbiota in *HLA-DRB1* shared epitope-positive participants, compared with shared epitope-negative controls in the SCREEN-RA cohort In all participants, *Prevotella_7* showed the most substantial positive log-fold change of 5 (q=0·0348). Other genuses that showed lower but significant positive log-fold associations were *Veillonella* (q=0·035), *Streptococcus* (q=0·035), *Ruminococcaceae UBA1819* (q=0·035), and *Coprobacter* (q=0·0020). In participants without symptoms associated with rheumatoid arthritis (excluding tender or swollen joints; grey shading), *Prevotella_7* solely remained positively associated with shared epitope positivity (q=0·0011).

In the SCREEN-RA cohort, *Prevotella*_*9* was positively associated with preclinical rheumatoid arthritis (q=0·021; figure 3). *Prevotella*_*7* was associated with HLA-DRB1 shared-epitope risk alleles for rheumatoid arthritis in the SCREEN-RA cohort (n=133; q=0·035); the association was stronger in a subgroup analysis in which 44 participants with swollen joints were removed to isolate genotype from rheumatoid arthritis pathophysiology (n=89; q=0·0011; figure 4). In the subgroup analysis of differential abundance against shared-epitope positivity in asymptomatic participants only, *Prevotella_7* was the only remaining taxon association. The key implication is that *Prevotella_9* and *Prevotella_7* are distinct from one another, and *Prevotella_9* is predicted to be *P copri.*^16^, ^19^

**Figure 5:**
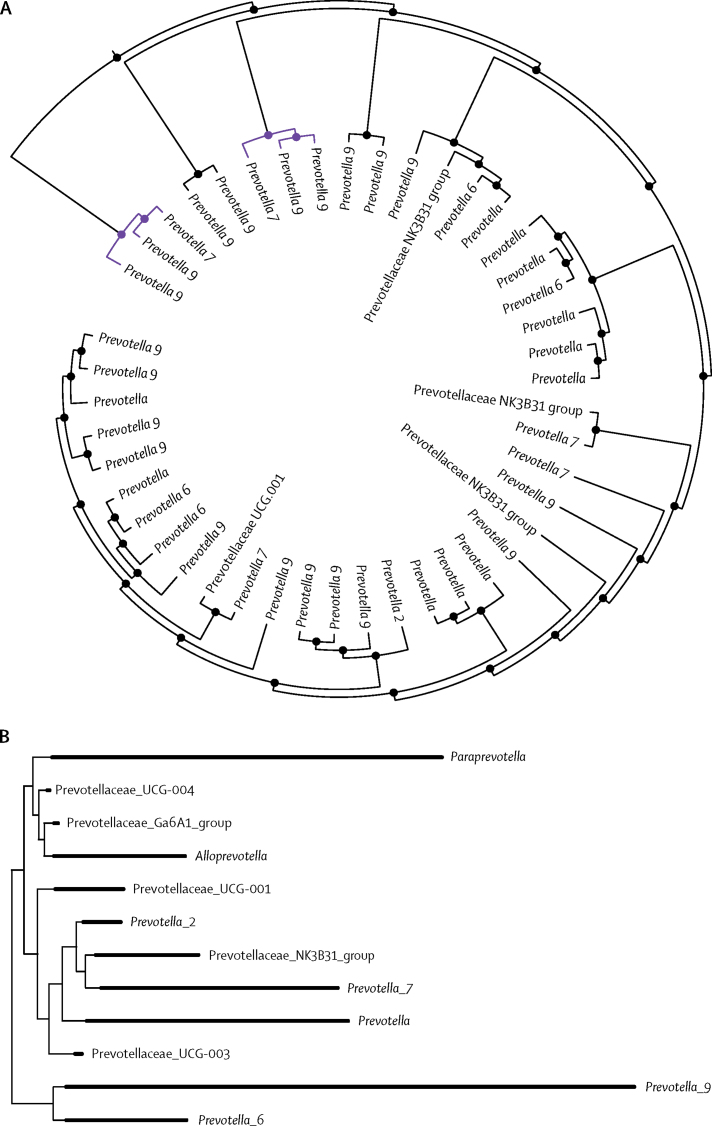
Relationship between *Prevotella* ASVs within stool samples from TwinsUK participants (A) Cluster tree showing an ecological community relationship between *Prevotella* ASVs within stool samples from TwinsUK participants. Taxa clades represent members of the same microbial ecological community. *Prevotella_9* and *Prevotella_7* are the only two taxon clusters present, and ASVs assigned to these taxa cluster in the same clade more frequently than do those in any other taxa. Clustering of *Prevotella_7* and *Prevotella_9* ASVs (shown in purple) indicates that the abundance of these taxa is interdependent; a biological interdependence and environmental niche similarity is suggested. Incremental levels are shown, from individual ASVs joined as two-taxon clusters, to the full set of Prevotellaceae ASVs joined by the final head node. (B) Phylogenetic tree for Prevotellaceae ASVs within TwinsUK participants. *Prevotella_9* and *Prevotella_7* are phylogenetically distinct from each other. ASV=amplicon sequence variant.

As *Prevotella* spp were the strongest taxon associations in our results and of particular interest in rheumatoid arthritis, we further investigated the phylogenetic and community relationships within Prevotellaceae. In particular, we were interested in the relationship between *Prevotella_7* (which we identified to be associated with genetic risk) and *Prevotella_9* (which we identified to be associated with preclinical rheumatoid arthritis). Cluster analysis showed a community relationship between *Prevotella* spp. *Prevotella_7* and *Prevotella_9* were the only two species clusters identified within the Prevotellaceae family, and they clustered together more frequently than any other group did, suggesting an interdependent community relationship between these taxa (figure 5). Visualisation of the Prevotellaceae phylogenetic tree showed that *Prevotella_7* and *Prevotella_9* are phylogenetically distinct from one another (figure 5B).

*Prevotella_7* might be implicated in rheumatoid arthritis pathogenesis, and might be increased in patients with rheumatoid arthritis (appendix p 1). Conversely, given that the rheumatoid arthritis-unaffected TwinsUK participants were beyond the mean age of rheumatoid arthritis onset, yet some had high genetic risk scores, these results might hypothetically reflect participants being resilient to the development of rheumatoid arthritis. Correspondingly, the genetic risk and microbiota associations showed might potentially be microbial markers of rheumatoid arthritis resilience. In this instance, the ratio of *Prevotella_7* to *Prevotella_9 (P copri)* would be expected to be higher in unaffected twins compared with rheumatoid arthritis-affected twins.

The relative abundance of *Prevotella* spp was calculated in 18 monozygotic female rheumatoid arthritis-discordant twin pairs who were excluded from our previous analysis, in whom rheumatoid arthritis diagnosis had been confirmed during clinical visit. A higher relative abundance of *Prevotella*_7 was found in the rheumatoid arthritis-affected twins compared with unaffected control twins (relative abundance 0·005 *vs* 0·001; appendix p 2). This finding neither supports nor rejects the hypothesis that *Prevotella_7* is implicated in rheumatoid arthritis aetiology. Abundance of *Prevotella* spp was not significantly associated with rheumatoid arthritis. The proportion of *Prevotella_7* (species prediction uncertain) to *Prevotella_9* (predicted *Prevotella copri*) was higher in the rheumatoid arthritis-affected twins than in the control twins (0·4 *vs* 0·01), suggesting that *Prevotella_7* is not a marker of rheumatoid arthritis resilience.

## Discussion

Our results show a link between host genetic risk for rheumatoid arthritis and the gut microbiota in two large cohorts. We found associations between gut microbiota and genetic risk in the absence of clinically detectable disease in TwinsUK. The strongest association between rheumatoid arthritis genetic risk and the gut microbiota was an increase in abundance of *Prevotella_7.* This finding was validated in the SCREEN-RA cohort, in which *Prevotella_7* was positively correlated with shared-epitope positivity. *Prevotella_7* was shown to share a biological interdependence with *Prevotella_9* (predicted *P copri*).

Considerable interest exists in *P copri* as a potential mediator of rheumatoid arthritis pathology, and the bacteria is a candidate keystone taxon enriched in the gut microbiota of patients with newly diagnosed rheumatoid arthritis. Since this observation was made, the human immune response to this microbe has been of considerable interest. Antibodies to *P copri* have been shown to associate with disease severity and T helper type 1 (Th1) cell-mediated and Th17 cell-mediated immune responses in patients with rheumatoid arthritis.^22^ Functional work has suggested a role for *P copri* in Th17 cell differentiation.^23^ That *P copri* is associated with new-onset rheumatoid arthritis before treatment with disease-modifying antirheumatic drugs,^4^ and is also associated with other inflammatory conditions,^24^ has fuelled speculation that inflammation is a prerequisite for *P copri* proliferation within the gut, relative to other taxa.^22^, ^25^ *P copri* might have adapted to thrive in a pro-inflammatory environment, and might further promote the inflammatory milieu, thereby enhancing the bacteria's own favoured environmental niche.^25^ In doing so, *P copri* is suggested to contribute to rheumatoid arthritis pathology. According to this model, a human–microbiota interspecies positive feedback loop is proposed. Another example of such a model is the hijacking of complement cascade by *Porphyromonas gingivalis.*^26^

An increase in abundance of three other taxa (*Ruminococcaceae_UCG-14*, *Rikenella,* and *Shigella*) with the PRS for rheumatoid arthritis was also observed. Ruminococcaceae and *Shigella* have been shown previously to be in higher abundance in rheumatoid arthritis patients compared with controls.^3^, ^4^ The evidence for a role of these taxa in rheumatoid arthritis is much weaker than for *P copri*, with lower replication across studies, and no functional link reported to date. However, the association both with patients with rheumatoid arthritis and with genetic risk for rheumatoid arthritis in a large, cohort without disease is interesting, and merits further investigation.

Although causality is not knowable from a cross-sectional association study, our results provide robust evidence indicating that host genetic factors influence the abundance of *Prevotella* in the gut micobiota. Both TwinsUK and SCREEN RA cohorts were balanced for ACPA positivity in relation to rheumatoid arthritis genetic risk loci. A previous study of patients with new-onset rheumatoid arthritis and healthy participants reported an inverse association of HLA genotype with abundance of *P copri*, in the opposite direction to that expected.^5^ This association might potentially relate to population differences or confounding factors that were not examined. The present study, using ASVs in a large sample without disease, provides more substantial evidence for an association of genotype with the rheumatoid arthritis gut microbiota.

Our findings suggest that the microbiota are altered before disease, which is in accordance with previous reports of *Prevotella* spp in patients with pre-rheumatoid arthritis^8^ and rheumatoid arthritis.^5^ Indeed, we found similar results in the patients with preclinical rheumatoid arthritis in first-degree relatives of patients with rheumatoid arthritis. In a study of the SCREEN-RA cohort,^8^ an enrichment of Prevotellaceae in the gut microbiota in patients with pre-rheumatoid arthritis was observed. In that study,^8^ no specific Prevotellaceae genera were shown to drive the association. Therefore, in this study we reanalysed these data using the more recent method of ASVs. ASVs offer an updated approach to traditional clustering based methods generating operational taxonomic unit (OTUs) from 16S sequences. As opposed to grouping sequences on the basis of a similarity threshold as for OTUs, the error rate is used to infer the original biological sequence and produce units of matched sequence. Therefore, ASVs offer higher resolution and have greater biological relevance than OTUs.^27^ The ASV analysis showed a false discovery rate-adjusted significant *Prevotella* genus association with a species-level group annotation, indicating potential *P copri*. Our follow-up investigation of patients with pre-rheumatoid arthritis revealed a further six novel genus associations, which had not been evident in the original OTUs*—Lactobacillus, Enterococcus, Faecalibacterium,* Ruminococcaceae UBA1819*, Veilllonella,* and *Butyrivibrio.* Of the six associations, the first four have been reported to be associated with rheumatoid arthritis,^4^, ^28^, ^29^ and Ruminococcaceae were associated with rheumatoid arthritis PRS in the TwinsUK cohort. *Butyrivibrio* has not yet been associated with rheumatoid arthritis, but is a physiologically interesting taxa associated with the production of short chain fatty acid and host metabolism.

This study has several limitations. First, in the pre-rheumatoid arthritis follow-up analysis both cases and controls were first degree relatives of patients with rheumatoid arthritis, with increased genetic risk compared with the general population. Patients with preclinical rheumatoid arthritis could have had higher genetic risk than the controls did, but because full genotyping was not available, we were unable to confirm this possibility. The TwinsUK cohort is predominantly female. The SCREEN-RA cohort is slightly more balanced in terms of sex; however, population prevalence of rheumatoid arthritis is much higher in women than men. Because the age of onset of rheumatoid arthritis is 30–65 years,^30^ the TwinsUK participants, who have a median age of 63 years, are less likely to develop disease than are the SCREEN-RA participants. However, the age of the cohort is of benefit to the design of our study of genetically high-risk yet unaffected individuals and helps us to understand microbiota differences in the absence of disease.

This study highlights the value of using the newer methods of ASVs, which can detect taxonomic variation overlooked by OTU-based methods. Further microbiota associations with rheumatoid arthritis are to be anticipated from improvements in sequencing and interpretation. Annotation of ASVs with the SILVA database^16^, ^17^ showed differences at the species level, which would be overlooked using other reference databases. Finally, modelling of the community relationship provides valuable insight into the underlying biology. Future studies should take advantage of these methods.

Taken together, these results support the hypothesis that microbiota is altered in individuals with genetic predisposition to rheumatoid arthritis before the onset even of preclinical rheumatoid arthritis. Our findings shed light on our understanding of microbiota in rheumatoid arthritis and addresses the issue of cause versus consequence—if microbial alteration precedes disease, the microbiota might lie on the causal pathway. However we cannot yet exclude the possibility that *Prevotella* spp are bystanders. Additionally, having genetic risk for rheumatoid arthritis and *Prevotella* spp is likely not sufficient for disease development, but rather might be one of several insults contributing to progression of rheumatoid arthritis pathology, in line with the favoured two-hit hypothesis of rheumatoid arthritis pathogenesis.^31^ The identification of preclinical rheumatoid arthritis represents an important clinical target in early disease intervention and is the subject of multiple immune-modulating clinical trials. Further, the genetic risk–microbiota associations that we identified might be applicable to other diseases because a crossover exists in genetic cause between rheumatoid arthritis and other autoimmune conditions. Finally, the microbiota might offer the opportunity for modulation of pre-disease pathways alone or in combination with immune-modulating drugs.

In conclusion, gut microbiota abundance is associated with the genotype for rheumatoid arthritis risk even in the absence of disease. Genotype might mediate key taxonomic associations of the gut microbiota with rheumatoid arthritis, particularly *Prevotella* spp,^4^, ^5^, ^22^, ^23^ suggesting that these species play a role early in the development of rheumatoid arthritis.

## Data sharing
