# Correction to *Lancet Rheumatol* 2020; 2: e418–27

**DOI:** 10.1016/S2665-9913(25)00098-0

**Published:** 2025-03-27

*Wells P M, Adebayo A S, Bowyer R C E, et al. Associations between gut microbiota and genetic risk for rheumatoid arthritis in the absence of disease: a cross-sectional study.* Lancet Rheumatol *2020; **2:** e418–27*—In this Article, references to the association between *Prevotella* spp and carrying established shared epitope risk alleles in the SCREEN-RA participants should have read “inversely associated”. Text in the summary, results, and discussion sections have been updated. In figure 4, the x axis has been reversed and the legend has been updated accordingly. These corrections have been made as of March 27, 2025.

