# Supplementary material for: Associations between gut microbiota and genetic risk for rheumatoid arthritis in the absence of disease: a cross-sectional study
Source: Lancet Rheumatol. 2020 Jun 25;2(7):e418–27. doi: 10.1016/S2665-9913(20)30064-3 (PMC7729822; doi:10.1016/S2665-9913(20)30064-3)
Supplement: Supplementary appendix [file mmc1.pdf]

THE LANCET
Rheumatology

Supplementary appendix

This appendix formed part of the original submission and has been peer reviewed. We post it as supplied by the authors.

Supplement to: Wells PM, Adebayo AS, Bowyer RCE, et al. Associations between gut microbiota and genetic risk for rheumatoid arthritis in the absence of disease: a cross-sectional study. *Lancet Rheumatol* 2020; **2:** e418–27.

Supplementary Material

Supplementary Methods

	SE Positive (n=74)	SE Negative (n=55)	T Test
**Age Median (IQR)**	56.5 (12.75)	57 (18.5)	0.69
**Female Sex n (%)**	62 (84)	47 (85.5)	0.79
**BMI Median (IQR)**	24.5 (4.8)	24.4 (4.22)	0.47
**ACPA positivity n (%)**	20 (27)	16 (29)	0.79

Supplementary Table 1. Age, sex, BMI and ACPA positivity in Shared Epitope (SE) positive and SE negative groups in the SCREEN-RA cohort. These factors were not significantly different between the case (SE positive) and control (SE negative) groups, and may therefore be described as statistically balanced.

Resilience hypothesis: abundance of *Prevotella spp*. in RA discordant twin pairs in TwinsUK cohort.

To be confident that the *Prevotella spp* findings in non-RA individuals with genetic risk for RA were not reflecting a "resilience-associated" microbiota (i.e. protective factors against developing disease despite having high genetic risk), we calculated the abundance of these species in 19 monozygotic RA discordant female twin pairs from TwinsUK, which had been excluded from the previous analysis because of the presence of disease. If associated with resilience we would expect the *Prevotella spp* to be preferentially expressed in the unaffected twin of each discordant pair. RA diagnosis had been confirmed during a clinical visit to a Rheumatologist or RA-trained research assistant. ASVs were generated as above and relative abundance of *Prevotella spp.* in RA discordant twin pairs calculated using the Phyloseq R package[19] (**Supplementary Figure 1**).

Supplementary Results

Alpha diversity	Estimate	Std. Error	R2	P
**Observed**	-0.011	0.021	0.251	0.6
**Shannon**	-0.007	0.024	0.026	0.76
**Simpson**	-0.023	0.034	0.006	0.41
**Faith's**	-0.009	0.021	0.116	0.65

Supplementary Table 2. Association of the RA PRS with alpha (within sample) diversity of the gut microbiota in TwinsUK participants. An association of RA PRS with alpha diversity was not demonstrated.

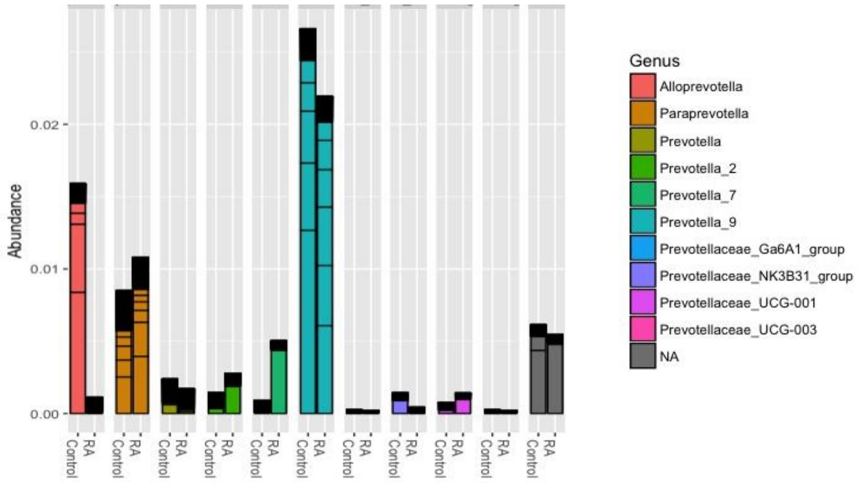

Supplementary Figure 1. Relative abundance of *Prevotella spp.* In 30 RA-discordant twin pairs from TwinsUK (total n = 60). The RA affected participants comprising 1 member of each twin pair were established (long-term treated) RA patients. *Prevotella_7* was more abundant in RA affected co-twins, compared to non-RA co-twins. No statistically significant differences in abundance of *Prevotella spp.* between RA discordant twin pairs were demonstrated.

Supplementary Table 1. SNPs included in the RA PRS.

Chromosome	SNP	A1	A2	P	OR	Replicated
1	rs624988	T	C	8.00E-10	1.09	No
1	rs840016	C	T	2.00E-06	1.11	No
1	rs923880	C	T	8.00E-06	0.25	No
1	rs72717009	T	C	5.00E-07	1.12	Yes
1	rs2317230	T	G	2.00E-07	1.07	No
1	rs6593803	T	C	2.00E-07	3.13	No
1	rs2228145	A	C	4.00E-09	1.08	Yes
1	rs12565755	C	T	5.00E-08	0.39	No
1	rs2105325	C	A	0	1.12	Yes
1	rs12140275	A	T	4.00E-09	1.11	Yes
1	rs3890745	T	C	1.00E-07	0.89	Yes
1	rs28411352	T	C	0	1.11	Yes
1	rs2301888	G	A	0	1.19	Yes
1	rs12131057	G	A	4.00E-07	1.16	No
1	rs2476601	A	G	2.00E-11	1.72	Yes
1	rs6679677	A	C	0	1.79	Yes
1	rs11121380	C	A	5.00E-08	11.11	No
1	rs998731	C	T	3.00E-09	1.11	Yes
2	rs6732565	A	G	9.00E-09	1.1	Yes
2	rs11676922	T	A	0	1.12	Yes
2	rs10865035	A	G	2.00E-06	1.12	No
2	rs9653442	C	T	0	1.11	Yes
2	rs11900673	T	C	1.00E-08	1.11	No
2	rs13385025	A	G	9.00E-07	1.11	No
2	rs6715284	G	C	3.00E-09	1.15	Yes
2	rs1980422	C	T	0	1.12	Yes
2	rs3087243	G	A	1.00E-08	1.15	Yes
2	rs231735	G	T	6.00E-09	1.17	No
2	rs12617656	T	C	0	1.557	Yes
2	rs41005	T	C	4.00E-06	0.27	No
2	rs1406428	T	C	2.00E-07	2.74	No
2	rs10175798	A	G	1.00E-09	1.08	Yes
2	rs13031237	T	G	8.00E-07	1.13	No
2	rs13017599	A	G	0	1.21	No
2	rs34695944	C	T	0	1.13	Yes
2	rs4305317	T	A	2.00E-06	1.45	No
2	rs13015080	A	G	3.00E-06	0.28	Yes
2	rs934734	G	A	5.00E-10	1.13	Yes
2	rs1858037	T	A	0	1.11	Yes
2	rs7574865	T	G	2.00E-06	1.17	Yes
2	rs11889341	T	C	0	1.13	Yes
3	rs73081554	T	C	5.00E-08	1.18	No
3	rs2062583	G	T	2.00E-06	0.63	No
3	rs9860428	C	A	4.00E-07	1.6	Yes
3	rs3806624	G	A	9.00E-09	1.08	Yes

3	rs9826828	A	G	9.00E-10	1.44	No
3	rs6774280	C	T	9.00E-07	5.5	No
3	rs4452313	T	A	5.00E-11	1.11	Yes
3	rs7624766	G	A	4.00E-07	2.28	No
3	rs13315591	C	T	5.00E-08	1.29	No
3	rs1809529	C	T	3.00E-07	0.28	No
4	rs2867461	A	G	0	1.13	No
4	rs11933540	C	T	0	1.15	No
4	rs13142500	C	T	2.00E-06	1.1	Yes
4	rs13119723	A	G	7.00E-07	1.12	No
4	rs45475795	G	A	4.00E-06	1.14	No
4	rs11937061	T	G	2.00E-07	6.7	No
4	rs3816587	C	T	9.00E-06	1.09	No
4	rs874040	C	G	0	1.19	No
4	rs6448432	A	G	4.00E-07	1.19	No
4	rs13137105	G	A	9.00E-07	6.39	No
4	rs2664035	A	G	3.00E-08	1.08	Yes
5	rs7731626	G	A	0	1.21	No
5	rs255758	C	A	7.00E-06	1.42	No
5	rs26232	C	T	4.00E-08	0.88	No
5	rs2561477	G	A	2.00E-11	1.11	Yes
5	rs1498103	A	T	6.00E-06	0.26	No
5	rs12519788	G	A	5.00E-08	1.82	Yes
5	rs657075	A	G	3.00E-10	1.12	Yes
5	rs6859219	C	A	1.00E-11	1.28	No
5	rs12109285	C	T	1.00E-07	130.2	No
5	rs1502644	C	A	1.00E-08	2.33	No
5	rs4336372	C	T	1.00E-06	0.65	Yes
5	rs4921283	A	G	1.00E-07	1.0752687	No
6	rs6568431	A	C	1.00E-07	1.14	No
6	rs9372120	G	T	8.00E-10	1.11	Yes
6	rs3093024	A	G	0	1.19	No
6	rs3093023	A	G	2.00E-11	1.11	Yes
6	rs1571878	C	T	0	1.17	Yes
6	rs1854853	A	G	0	1.4164	Yes
6	rs12529514	C	T	2.00E-08	1.14	No
6	rs3763309	A	C	0	2.3	No
6	rs6457617	T	C	1.00E-09	2.36	Yes
6	rs9275406	T	G	0	2.1	No
6	rs660895	G	A	0	3.62	No
6	rs7765379	G	T	0	2.51	No
6	rs13192471	G	A	0	1.97	No
6	rs6910071	G	A	0	2.88	No
6	rs9378815	C	G	2.00E-10	1.09	Yes
6	rs284515	G	A	7.00E-07	0.35	No
6	rs284511	C	T	3.00E-08	0.41	No
6	rs2233434	G	A	0	1.19	Yes

6	rs2233424	T	C	0	1.24	Yes
6	rs9373594	T	C	3.00E-09	1.09	Yes
6	rs2451258	T	C	3.00E-11	1.1	Yes
6	rs6920220	A	G	0	1.22	Yes
6	rs5029924	T	C	0	1.74	No
6	rs7752903	G	T	0	1.38	Yes
6	rs10499194	C	T	1.00E-09	1.33	No
6	rs2230926	C	A	2.00E-06	1.31	No
6	rs75908454	C	T	6.00E-07	0.42	No
7	rs42041	G	C	4.00E-06	1.11	No
7	rs4272	G	A	5.00E-09	1.1	Yes
7	rs6956740	T	C	5.00E-07	3.49	No
7	rs11761231	T	C	4.00E-07	1.44	No
7	rs10488631	C	T	4.00E-11	1.16	No
7	rs3807306	T	G	3.00E-07	1.44	No
7	rs67250450	T	C	3.00E-09	1.11	Yes
7	rs12531711	G	A	0	1.45	No
8	rs1600249	T	A	5.00E-06	0.77	No
8	rs2736340	T	C	1.00E-11	1.21	Yes
8	rs2736337	C	T	0	1.11	Yes
8	rs678347	G	A	7.00E-09	1.1	Yes
8	rs16938910	T	C	4.00E-07	28.66	No
8	rs800586	A	G	2.00E-07	9.78	No
8	rs1516971	T	C	3.00E-11	1.16	Yes
9	rs11574914	A	G	0	1.13	Yes
9	rs2812378	G	A	3.00E-08	1.12	No
9	rs951005	A	G	4.00E-10	1.19	No
9	rs12379034	G	A	0	1.489	Yes
9	rs1329568	T	G	8.00E-07	19.64	No
9	rs9299346	A	G	2.00E-06	2.76	No
9	rs2072438	T	C	3.00E-09	1.142	No
9	rs3761847	G	A	0	1.32	Yes
9	rs10985070	C	A	5.00E-11	1.08	Yes
10	rs10821944	G	T	0	1.16	No
10	rs71508903	T	C	0	1.16	Yes
10	rs3824660	C	T	3.00E-09	1.1	Yes
10	rs1679568	A	G	8.00E-07	0.35	Yes
10	rs706778	T	C	1.00E-11	1.14	Yes
10	rs2104286	C	T	1.00E-06	1.19	No
10	rs12413578	C	T	8.00E-08	1.2	Yes
10	rs16906916	C	A	8.00E-07	14	No
10	rs12570744	T	C	2.00E-07	0.49	No
10	rs4750316	G	C	4.00E-06	0.91	Yes
10	rs947474	A	G	3.00E-10	1.12	Yes
10	rs3125734	T	C	5.00E-09	1.2	No
10	rs6479800	C	G	4.00E-08	1.11	Yes
10	rs726288	T	C	9.00E-09	1.22	No

10	rs2671692	A	G	3.00E-09	1.07	No
10	rs793108	T	C	1.00E-09	1.08	Yes
11	rs508970	A	G	3.00E-06	1.07	No
11	rs4409785	C	T	1.00E-11	1.12	Yes
11	rs10790268	G	A	0	1.17	Yes
11	rs73013527	C	T	1.00E-10	1.09	Yes
11	rs4937362	T	C	8.00E-07	1.09	No
11	rs968567	C	T	2.00E-08	1.12	No
11	rs518167	A	G	2.00E-06	2.24	No
11	rs7940423	A	G	1.00E-07	10.9	No
11	rs72991	C	T	5.00E-07	2.45	No
11	rs1079467	C	G	2.00E-06	0.29	No
11	rs3781913	T	G	6.00E-10	1.12	No
11	rs331463	T	A	1.00E-07	1.12	Yes
12	rs773125	A	G	1.00E-10	1.09	Yes
12	rs1633360	T	C	7.00E-08	1.08	Yes
12	rs11051970	T	G	1.00E-06	2.08	No
12	rs1678542	C	G	1.00E-07	1.2	Yes
12	rs11045392	C	T	2.00E-06	3.3	No
12	rs3184504	T	C	6.00E-06	1.08	No
12	rs10774624	G	A	7.00E-09	1.09	No
12	rs3794271	G	A	4.00E-06	3.2	No
12	rs12831974	C	T	6.00E-06	1.27	No
13	rs9557321	C	T	6.00E-08	1.73	No
13	rs9603612	G	C	0	1.098901	No
13	rs9603616	C	T	0	1.1	Yes
13	rs4942242	T	C	2.00E-07	0.13	No
13	rs9604529	G	A	7.00E-07	0.35	No
13	rs3790022	A	G	1.00E-06	1.4925	No
14	rs2582532	C	T	5.00E-08	1.18	Yes
14	rs7155603	G	A	1.00E-07	1.16	No
14	rs3783637	C	T	2.00E-06	1.1	No
14	rs17118552	G	A	2.00E-07	17.19	No
14	rs1624005	G	A	7.00E-06	2.59	No
14	rs7141276	A	G	2.00E-06	0.37	No
14	rs2841277	T	C	0	1.15	No
14	rs1957895	G	T	4.00E-07	1.09	No
14	rs3783782	A	G	2.00E-09	1.14	Yes
14	rs1950897	T	C	8.00E-11	1.1	Yes
15	rs1914816	A	G	7.00E-07	0.87	No
15	rs1898036	C	T	2.00E-07	7.73	No
15	rs7164176	G	A	5.00E-07	0.28	No
15	rs17374222	A	C	2.00E-06	1.13	No
15	rs8026898	A	G	0	1.15	Yes
15	rs12901682	A	C	4.00E-08	135.02	No
15	rs8032939	C	T	0	1.13	Yes
15	rs6496667	A	C	1.00E-06	1.09	No

16	rs6500395	C	T	6.00E-07	2.2	No
16	rs2280381	T	C	2.00E-06	1.12	No
16	rs13330176	A	T	9.00E-09	1.12	Yes
16	rs16973500	T	NA	9.00E-06	0.24	No
16	rs7404928	T	C	4.00E-06	1.08	No
16	rs4780401	T	G	9.00E-09	1.09	Yes
17	rs72634030	A	C	2.00E-09	1.12	No
17	rs2872507	A	G	9.00E-07	1.1	No
17	rs1877030	C	T	2.00E-08	1.09	No
17	rs12601925	G	A	6.00E-09	2.15	Yes
18	rs2469434	C	T	9.00E-10	1.07	Yes
18	rs16977065	T	C	1.00E-07	33.72	No
18	rs2847297	G	A	2.00E-08	1.1	No
18	rs8083786	G	A	0	1.14	Yes
18	rs6506569	T	C	7.00E-06	1.98	No
18	rs2002842	A	C	6.00E-06	1.15	No
18	rs1943199	T	G	2.00E-08	1.94	No
19	rs7258015	C	T	3.00E-08	1.18	No
19	rs1273516	A	G	9.00E-07	4.26	No
19	rs1543922	T	C	3.00E-07	34.48	No
19	rs34536443	G	C	0	1.46	No
20	rs4810485	G	T	8.00E-09	0.91	No
20	rs4239702	C	T	0	1.13	Yes
20	rs6138892	C	T	3.00E-07	10.93	No
20	rs6026990	A	T	6.00E-07	0.63	No
21	rs2075876	A	G	4.00E-09	1.18	No
21	rs73194058	C	A	3.00E-08	1.13	Yes
21	rs2837960	G	T	2.00E-06	1.05	No
21	rs8133843	A	G	3.00E-09	1.08	Yes
21	rs11203203	A	G	4.00E-06	1.11	No
21	rs1893592	A	C	1.00E-11	1.11	No
22	rs1043099	C	G	7.00E-09	1.19	No
22	rs3218251	A	T	6.00E-06	1.08	No
22	rs743777	G	A	1.00E-06	1.09	No
22	rs909685	A	T	0	1.13	Yes
22	rs2283790	G	A	2.00E-11	1.22	No
22	rs11089637	C	T	2.00E-09	1.08	Yes
